# Racial Disparities in Opioid Overdose Deaths in Massachusetts

**DOI:** 10.1001/jamanetworkopen.2022.9081

**Published:** 2022-04-28

**Authors:** Che-Yi Liao, Gian-Gabriel P. Garcia, Catherine DiGennaro, Mohammad S. Jalali

**Affiliations:** 1 Georgia Institute of Technology, Atlanta; 2 Massachusetts General Hospital Institute for Technology Assessment, Harvard Medical School, Boston; 3 Sloan School of Management, Massachusetts Institute of Technology, Cambridge

## Abstract

This cross-sectional study evaluates publicly available data from Massachusetts on reporting trends in opioid-related overdose deaths by race and ethnicity.

## Introduction

Since 2013, opioid-related overdose deaths have risen disproportionately among US non-Hispanic Black and Hispanic people.^1,2,3^ Timely detection and policy response to these disparities require monitoring systems to signal emerging changes in overdose trends.^4^ To this end, the US Centers for Disease Control and Prevention reports provisional data on drug overdose deaths^5^ that can be disaggregated by state and substance, facilitating the identification of emerging trends in substance involvement. However, these data lack demographic detail, making tracking of trends in overdose mortality by race and ethnicity challenging. Massachusetts is one of the few states that provides individual-level death data with 3 key characteristics: a relatively short lag of 3 to 4 months in certifying official cause of death, detailed demographic information, and substance-specific underlying cause of death information.^6^ Therefore, data from Massachusetts can be used to illustrate the limitations of a failure to report granular opioid mortality data. Accordingly, we investigated racial and ethnic disparities in excess opioid-related overdose deaths during the COVID-19 pandemic in Massachusetts.

## Methods

In this cross-sectional study, we used individual-level death records between January 1, 2017, and March 1, 2021, from the Massachusetts Registry of Vital Records and Statistics to identify decedents with opioid-related overdose as their underlying cause of death and pulled demographic data on their racial and ethnic background (American Indian, Asian, Black, Hispanic, and White). The Mass General Brigham Institutional Review Board determined this study exempt from review, and the need for informed consent was waived because data were not obtained through interaction or intervention with study participants, nor was identifiable private information about living individuals obtained per their Definition of Human-Subjects Research. This study followed the STROBE reporting guideline.

To calculate excess mortality during the COVID-19 pandemic, we fitted an autoregressive integrated moving average model on data up to February 29, 2020, to project the number of opioid-related overdose deaths from March 1, 2020, to March 1, 2021. We then subtracted the actual number of deaths from these projected figures. Data were analyzed with R Language, version 4.1.2 (The R Foundation for Statistical Computing); statistical significance was not assessed.

## Results

Between March 1, 2020, and March 1, 2021, there were 2103 recorded opioid-involved deaths in Massachusetts, including 33 American Indian, 20 Asian, 186 Black, 299 Hispanic, and 1565 White people (1539 [73.2%] men; 564 [26.8%] women; mean [SD] age, 42.6 [12.4] years). The Figure illustrates cumulative excess mortality overall and disaggregated by race and ethnicity during this period.

**Figure.**
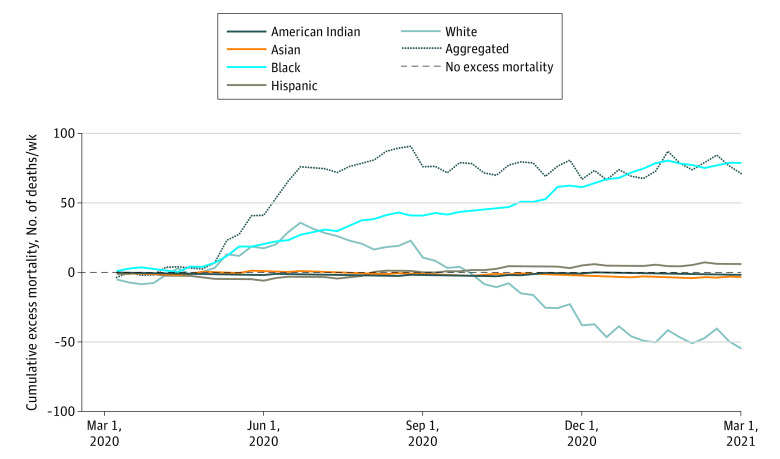
Cumulative Excess Mortality The cumulative excess mortality of aggregated opioid-related decedents without racial and ethnicity information and that of selected racial and ethnic groups. The horizontal dotted line at 0 denotes the situation when the projections perfectly fit the actual number of opioid-related overdose deaths.

Of note, we observed an increase in cumulative excess mortality overall. Disaggregated by race and ethnicity, however, cumulative excess mortality for White people peaked in June 2020 at 35.7 deaths/wk and became negative after September 2020 but remained stable for American Indian, Asian, and Hispanic people. In contrast, cumulative excess mortality increased continuously for Black people. The magnitude of excess deaths among Black people may be associated with an exacerbation of pre–COVID-19 trends stemming from disproportionate harms at the intersection of the opioid crisis, COVID-19, and structural racism present in health care and law enforcement systems.^3^

## Discussion

In this cross-sectional study, we used publicly available data on opioid-related overdose mortality from Massachusetts as an example to discuss a broader issue at the national level: Policy makers and opioid researchers currently must choose either timely provisional data without demographic information or detailed certified data with an 18- to 24-month lag. The disconnect between aggregated and disaggregated trends by race and ethnicity in Massachusetts illustrates the importance of disaggregating opioid overdose mortality data.

Our analysis was limited to 1 state; however, national-level data for opioid-related overdose deaths with demographic detail are 18 to 24 months behind. As such, underlying disparities in opioid overdose trends may be hidden in many parts of the country, precluding timely warnings to the public and policy makers.

The findings from this cross-sectional study suggest that to facilitate timely assessments of widening racial disparities in opioid-related overdose deaths, states and the Centers for Disease Control and Prevention should consider releasing opioid-related overdose counts more rapidly, including race and ethnicity data. We implore national- and state-level policy makers to divert resources toward enhancing data reporting practices so that disproportionate harms may be adequately measured, evaluated, and addressed.
